# Investigating Medical Student's Preferences for Internet-Based Healthcare Services: A Best-Worst Scaling Survey

**DOI:** 10.3389/fpubh.2021.757310

**Published:** 2021-12-06

**Authors:** Richard Huan Xu, Ling-ming Zhou, Eliza Lai-yi Wong, Dong Wang

**Affiliations:** ^1^Department of Rehabilitation Sciences The Hong Kong Polytechnic University, Kowloon, Hong Kong SAR, China; ^2^JC School of Public Health and Primary Care The Chinese University of Hong Kong, Shatin, Hong Kong SAR, China; ^3^School of Health Management Southern Medical University, Guangzhou, China; ^4^Institute of Health Management Southern Medical University, Guangzhou, China

**Keywords:** best-worst scaling, preference, healthcare, medical student, Internet

## Abstract

**Objective:** This study aimed to investigate the importance of providing Internet-based healthcare services based on the preference of a sample of medical students in China.

**Methods:** An online best-worst scaling (BWS) survey with Case 1 design was conducted. Balanced independent block design generated 12 choice task profiles for each participant to answer. Descriptive analysis was used to describe the respondents' characteristics; Multinomial and mixed logit regression methods were used to investigate the importance of Internet-based services based on respondents' preferences.

**Results:** A total of 1,296 students completed the online survey and rated “Clinical Service,” “Decision Aids,” and “Public health” as the three most important services that should be provided through an Internet-based healthcare system. Providing “Medical Education” via the Internet was chosen as the least important service by the respondents. Subgroup analysis indicated that students studying clinical medicine and non-clinical medicine considered providing “Medical Education” and “Public Health,” respectively, as more important services than others.

**Conclusions:** This BWS study demonstrated that providing “Clinical Service,” “Decision Aids,” and “Public Health” through the Internet are the three most important services based on medical students' preferences in China. Further research is needed to investigate how to improve medical students' skills in using internet-based healthcare services in medical education programs.

## Introduction

Growing numbers of patients, families, and other interested stakeholders seek healthcare-related information on the Internet for a variety of purposes (1), such as evaluating their health, gathering knowledge about a newly diagnosed illness, or comparing the performance of different healthcare providers (2, 3). Previous studies have shown that an increasing number of people now trust Internet-based information and tend to make their health-related decisions based on that information—even before seeking professional advice (4–6). This may, thus, reshape the way in which medical providers interact with and provide consultations for their patients, and how they develop harmonious relationships with patients and their families. However, the fact that the preferences of medical students and their attitudes toward providing Internet-based healthcare services are rarely considered or embedded in current training programs may represent a challenge for our current medical education system.

The Internet is increasingly recognized as an effective way for improving the accessibility, equity, and cost-efficiency of healthcare for a wider range of patients with a varied set of preferences and needs (7–10). It enables healthcare providers to access information rapidly, make decisions based on the best clinical evidence, collect up-to-date information, and communicate with patients electronically and efficiently (11). Although the Internet supports a shift toward more patient-centered care, the idea of delivering healthcare services through the Internet is still open to challenge in areas such as providing intensive therapy, connecting the online, and offline services (12), and the controlling of costs incurred in maintaining the system (13). Internet-based healthcare is also an efficient way to optimize resource allocation and develop innovative service models. However, policy makers and medical professionals have to make a trade-off between the quantity and the quality of services that can be provided via the Internet based on each provider's limited capability, including both skills and budget.

In China, there are currently more than 800 million Internet users, accounting for 21% of users worldwide (14). The Internet has changed people's behaviors in relation to searching for healthcare information and services searching (15) but individual preferences regarding what kind of and how Internet-based healthcare services should be provided and how have rarely been discussed. In 2018, the State Council of China and the National Health Commission jointly decided to launch a nation-wide online service system in the next 10 years to promote the integration of healthcare areas, such as public health, privately contracted doctors, medical supplies, and medical insurance reimbursement settlement (16), but whether medical professionals equipped with sufficient knowledge and skills to provide web-based services and engage with patients in searching for and using the online information to maintain health is unknown. In particular, the outbreak of COVID-19 and subsequent mass lockdown has made the offering of web-based healthcare services more significant than ever before. However, although training on using the Internet to provide and streamline healthcare services is increasingly popular in medical education (17, 18), there is no general agreement on what types of training should be provided.

Medical students are an ideal population for studying the impact of internet-based healthcare services for two reasons. First, they are young people, and therefore likely to be near-constant users of the Internet. This means they are more likely to use the Internet to search for health-related information and services than the rest of the population. Second, as future medical providers, their attitudes toward Internet-based healthcare services would provide valuable information regarding the potential updating of medical training systems and the reform of online healthcare delivery services. Hence, the objective of this study was to help determine the relative importance of different Internet-based healthcare services by investigating the preferences of a sample of medical students in China.

## Methods

### Best-Worst Scaling Experiment

Best-Worst Scaling (BWS) is a method used to measure individuals' stated preferences, and it has increasingly been employed in the studies of health economics (19). BWS has been categorized into three types: Case 1 (object case), Case 2 (profile case), and Case 3 (multi-profile case). Case 1 is the simplest; individuals are repeatedly presented with a particular set of objects and asked to separately choose the most and the least attractive object from different combinations of selected objects. In Case 2, individuals are presented with a single profile that comprises different attributes with various levels and asked to choose one attribute–level combination as the best in that profile and another as the worst. Case 3 presents multiple profiles to the individuals, and they need to choose the best and worst ones in each choice set. The details of the BWS methodology are readily available (20). In this study, BWS Case 1 was adopted to investigate the participants' preferences in using Internet-based healthcare services.

BWS methods, especially the object and profile cases, have been increasingly used in health care in recent years (19, 21). Compared with the discrete choice experiment method, BWS is analytically less sophisticated. In China, the BWS method is widely used in business studies; however, its application in the area of health has been limited. For example, Xu et al. have used the BWS method to investigate the medical students' preferences about shared decision making with patients (22), while Mori and Tsuge reported on preferences regarding the adverse effects of tobacco use in China using the BWS method (23). The feasibility and acceptability of the BWS method have therefore been confirmed in Chinese studies.

### Identification of Attributes

A six-step process was adopted to develop the BWS questionnaire. First, a draft summary containing several attributes was drawn up by the research team based on the decree “Guideline of Development and Promotion of Providing Internet-based Healthcare Services in China” (16). Second, the authors reviewed the existing evidence concerning the barriers and facilitators that affect Internet usage to improve health outcomes (24–30). Another three possible attributes were added to the attribute pool. Third, three focus group interviews (involving nine doctors and seven nurses) were conducted to discuss the feasibility of included attributes until a consensus was reached. After the discussion, some attributes were revised and combined to create a set of 10 attributes. Fourth, the list of attributes was given to an expert panel (researchers, policy makers, and hospital managers) for further review. Subsequent discussion and review of national and regional documents, briefings, and reports were conducted, some words and expressions were revised, and a draft of nine attributes was confirmed. Fifth, to evaluate the validity of the attributes, the draft was given to a group of patients to seek their opinions based on a customer perspective, but no further revisions were made. Finally, the research team reviewed all the information and evidence from each of the previous steps, and a BWS questionnaire containing nine attributes (or services) was finally confirmed. The description of each attribute is listed in Table 1.

**Table 1:** Internet-based healthcare services attributes and explanations.

**Attribute**	**Description**
Clinical service	•Public hospitals provide safe and proper online clinical services and consultations to the patients with minor acute disease or chronic conditions
Public health	•Provide guidance on chronic condition management, information of vaccination and regular monitoring of pregnancy and providing associated information
Family doctor	•Family doctor gives online basic health care, including treating you when you are sick and encouraging preventative health care
Internet pharmacy	•Online providing prescription drugs of minor, acute and chronic diseases and deliver to you by courier
Customer eClaim	•Personal online medical insurance claim across different level of institutions or different geographical locations
Medical education	•Establishment of online platform providing online clinical and health promotion courses
Decision aids	•Online medical image processing, analysis and visualization; Online treatment of multiple disciplinary team; and the application of Artificial intelligence.
Data sharing	•Establishment of the online platform to share the data of diagnosis, test results and patients' electronic medical records across different levels of healthcare institutions
Monitoring & feedback	•Providing the online information of the performance of healthcare institutions; patient online complaint system and the online register system for hospitals and healthcare professionals

### Experiment Design

A balanced incomplete block design (BIBD) was used to construct the choice task. BIBD is the optimal way to create a design in which each attribute level is equally replicated and appears within a block with every other attribute level an equal number of times (31). A BIBD therefore ensures equal probabilities of selecting each attribute. The experiment design is shown in Table 2. A total of 12 choice task profiles were created, with three attributes each. For each choice task, a brief description of the background and attributes was provided. Participants were asked to select two attributes, of which one was the most important and the other was the least important service that the Internet-based healthcare system should provide. In total, each participant had to make 24 choices (12 best and 12 worst).

**Table 2:** The BIBD design of the BWS questionnaire.

**Attribute**	**Choice set (CS)**												**No. of appearances**
	**CS1**	**CS2**	**CS3**	**CS4**	**CS5**	**CS6**	**CS7**	**CS8**	**CS9**	**CS10**	**CS11**	**CS12**	
Attribute 1	1	0	0	1	0	0	1	0	1	0	0	0	4
Attribute 2	0	0	0	1	0	0	0	1	0	0	1	1	4
Attribute 3	0	0	0	0	1	0	0	0	1	1	0	1	4
Attribute 4	1	1	0	0	0	1	0	0	0	0	0	1	4
Attribute 5	0	1	0	0	1	0	1	1	0	0	0	0	4
Attribute 6	0	1	1	1	0	0	0	0	0	1	0	0	4
Attribute 7	0	0	0	0	0	1	1	0	0	1	1	0	4
Attribute 8	0	0	1	0	0	1	0	1	1	0	0	0	4
Attribute 9	1	0	1	0	1	0	0	0	0	0	1	0	4
No. of attributes in the choice set	3	3	3	3	3	3	3	3	3	3	3	3	-

**Figure 1:**
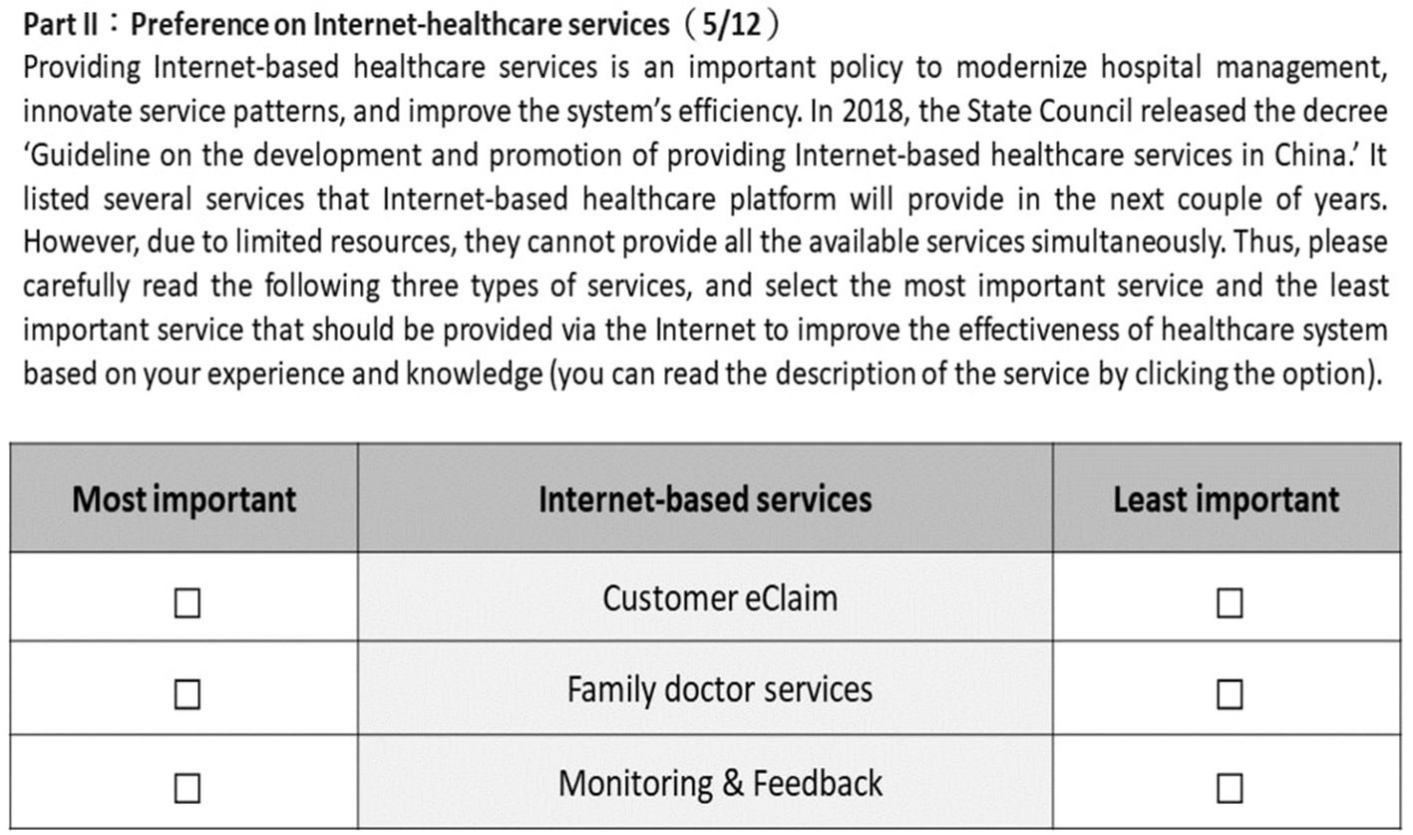
An example of the BWS questions.

Ten medical students were invited to participate in a pilot study. All of them were first asked to complete the questionnaire and a short interview was conducted immediately afterwards. During the interview, the participants were asked to express to the researchers their overall impression of the questionnaire, questions or concerns regarding the structure, misunderstandings with respect to the words or expressions, and advice on revising the questionnaire. After revising the terms and structure, the final BWS questionnaire was confirmed. An example of the choice set is shown in Figure 1.

There are different options regarding the optimal sample size for conducting a BWS survey. Lancsar and Louviere proposed that more than 20 respondents per choice set could estimate the reliable models (32) while a recent systematic review found that the sample size adopted in BWS Case 1 studies in the field of healthcare ranges between 15 and 803 (with the median being 175) (19). Thus, in this study, it was decided to use a minimum sample size of 500.

### Survey and Data Collection

This study was conducted in the Guangdong (GD) province, which has the largest economy in China and a population of around 110 million, and had a gross health expenditure of 461.9 billion Chinese Yuan (~67 billion US dollars) in 2017 (33). All the students from two medical universities were invited to participate in this study. The data were collected through a cross-sectional online survey in October/November, 2019. The officers in charge of student affairs from the two universities were approached first, and then, a brief description of the study and a link to the online survey were sent to all the students through WeChat, the most popular multipurpose messaging mobile app in China. The first page of the questionnaire was the informed consent page—the survey could not proceed until the participants read and clicked the “Agree” button at the end of that page. Our questionnaire comprised two sections: the first was the BWS questionnaire, which was developed based on the steps we described in the previous experiment design section; the second was a series of questions regarding respondents' personal information, such as gender, age, and the study program in which they were enrolled. Data quality was ensured based on completion time and missing rate. Based on the findings of the pilot study, we only use the data of respondents who completed the questionnaire ≥10 min, with no missing data and no abnormal response pattern reported for this analysis. This was an anonymous survey, no personal identifiable information was collected. The study protocol and informed consent were approved by the Institutional Review Board of the Third Affiliated Hospital of the Southern Medical University (Ref ID: 2019-044).

### Statistical Analysis

BWS response data were analyzed using two methods. First, count analysis as used to examine the choice frequency for each attribute. Three types of best-worst scores were presented.

1. BWS Score: This was calculated as the difference between the number of times an attribute was selected as the most important and the number of times that attribute was selected as the least important for each of the 12 attributes. If the BWS score is positive, the attribute was chosen more often as the most than as the least important, whereas a negative score indicated the reverse (34).
2. Scaled BWS Score: The Scaled BWS score was computed by dividing the total best score by the total worst score for each attribute and then calculating the square root of that result. The Scaled BWS score indicated the choice probability relative to the most important attribute (21).
3. Mean BWS Score: This was calculated as the BWS scores divided by the number of participants who responded to each attribute. All the BWS questions were predefined as compulsory in the online survey; thus, no missing data needed to be imputed in our analysis.

Then, two regression methods, a multinomial logit (MNL) and a mixed logit (MXL) model, were adopted to perform the data analysis. MNL and MXL incorporate the logit procedure (where each attribute has dual coding, best = 1 if the attribute is chosen as most important, and best = 0, otherwise; meanwhile, worst = 1 if the attribute is chosen as least important, and worst = 0, otherwise) to estimate propensity scores, describing the probability that an attribute is present in a specific combination of attributes (35). The structure of the relationship between the difference in utility between the best and worst ($U_{diff}^{i}$ on the latent utility scale) for choice task *i* (*i* = 1, 2, 3…13) and the 12 independent variables (attributes) was presented. The standard MNL and MXL functions link the observed discrete choice (0 or 1) with the estimated latent utility (36).


$$\begin{array}{l} {}*U_{diff}^{i}=\beta_{CS}D_{CS}^{i}+\beta_{PH}D_{PH}^{i}+\beta_{FD}D_{FD}^{i}+\beta_{IP}D_{IP}^{i}+s\beta_{CC}D_{CC}^{i}\\ \;+\beta_{ME}D_{ME}^{i}+\beta_{DA}D_{DA}^{i}+\beta_{DS}D_{DS}^{i}+\beta_{MF}D_{MF}^{i}+\varepsilon^{i} \end{array}$$

For MNL analysis, the coefficient of each attribute, which reflects the relative importance of that attribute compared to others, was calculated. Odds ratios were also presented to facilitate the explanation of the importance of attributes. MXL was used to explore the preference heterogeneity. It assumed that the parameters varied across individuals, and, therefore, accounted for the sample's heterogeneity (32). Along with the coefficients, the MXL model derived another standard deviation statistic to indicate unexplained variation around each attribute's mean. A standard deviation value significantly departing from zero indicated significant heterogeneity. Analysis of variance was used to conduct the heterogeneity analysis of mean BWS scores in two respondent subgroups (study year and program). The R software was used to design the BWS questionnaire and to perform all of the statistical tests (37). Statistical significance was set at *P* ≤ 0.05.

## Results

Table 3 presents the demographics of the respondents. A total of 1,296 students completed the online survey and provided valid responses. Nearly 75% of them were female, and the mean age was 20.01 years. Of the respondents, 96.2% were undergraduate students and only 2.6% indicated that they never search for any medical information on the Internet.

**Table 3:** The demographics of the sample (*n* = 1,294).

	***n***	**%**
**Sex**		
Male	320	24.7
Female	974	75.3
**Ethnicity**		
Han	1,139	88.0
Others	155	12.0
**Educational level**		
Undergraduate	1,245	96.2
Postgraduate	49	3.8
**Year in school**		
One	426	32.9
Two	266	20.6
Three	357	27.6
Four	178	13.8
Five	18	1.4
P-one^a^	29	2.2
P-two	10	0.8
P-three	10	0.8
**Program**		
Clinical medicine^b^	611	47.2
Non-clinical medicine	683	52.8
**Search health information online before visit doctor**		
Never	33	2.6
Few	231	17.9
Sometimes	491	37.9
Often	418	32.3
Always	121	9.4
	**Mean (SD)** ^c^	**Range**
Age	20.01 (3.59)	17–41
Health status	7.35 (1.51)	1–10
Satisfaction of last hospital visit	6.72 (1.88)	1–10
Trust online health information	5.42 (1.46)	1–10
Support to promote online health (0–10), mean (sd)	8.65 (1.52)	1–10

a *P-one, first year of postgraduate study*.

b *Clinical medicine included students studying clinical medicine, dentistry, traditional Chinese medicine, and nursing*;

c *SD, Standard deviation*.

Table 4 shows the results of the BWS survey. We found that respondents rated “Clinical Service” as the most important type of service that should be provided through an Internet-based healthcare system (Mean BWS score = 0.6, 95% Confidence Interval [C.I.] 0.49–0.72). Providing online “Medical education,” with a mean BWS score of −0.81 (95% C.I. −0.93 to −0.69), was chosen as the least important service. The relative importance of Internet-based healthcare services was also estimated by the MNL and MXL models. The importance of each attribute was estimated relative to the attribute of “Medical education,” which was consistently rated as the least important attribute (reference level). In both models, the results show that “Clinical Service” and “Decision Aids” were the services most favored by the respondents, while ‘Public Health’ and “Data Sharing” were the next most sought-after services. All the other attributes seemed to be of intermediary importance except for “Family doctor,” which was close to the reference attribute “Medical Education,” whereas the MXL model indicated that “Customer eClaim” and “Family doctor” were significantly less important than “Medical Education.” Moreover, the MXL model also provided the SD estimation for each attribute. The non-significant results reflected the absence of any substantial heterogeneity regarding the relative importance of each attribute existed (Table 5).

**Table 4:** The results of the BWS survey.

	**Best**	**Worst**	**BWS score**	**SD of BWS score^a^**	**Mean BWS score**	**95% C.I of mean BWS^b^**	**Scaled BWS score**	**SD of scaled BWS score**
Clinical service	2,109	1,328	781	0.15	0.6	0.49 to 0.72	1.26	1.0
Decision aids	2,135	1,380	755	0.14	0.58	0.47 to 0.69	1.24	0.99
Public health	1,849	1,276	573	0.11	0.44	0.33 to 0.55	1.2	0.95
Data sharing	1,995	1,547	448	0.09	0.35	0.23 to 0.47	1.14	0.9
Internet pharmacy	1,716	1,590	126	0.02	0.09	0 to 0.21	1.04	0.82
Monitoring & feedback	1,688	1,593	95	0.02	0.07	−0.05 to 0.19	1.03	0.82
Customer eClaim	1,397	2,117	−720	−0.14	−0.56	−0.69 to −0.63	0.81	0.64
Family doctor	1,351	2,358	−1,007	−0.19	−0.78	−0.91 to −0.65	0.76	0.6
Medical education	1,288	2,339	−1,051	−0.2	−0.81	−0.93 to −0.69	0.74	0.59

a *SD, Standard deviation*.

b *95% C.I., 95% confidence interval*.

**Table 5:** Results of multinomial logit regression and mixed logit regression analyses.

	**Multinomial logistic model**			**Mixed logit model**		
	**B^a^**	**SE^b^**	**OR^c^**	**b**	**SE**	**SD^d^**
Clinical service	0.94^***^	0.03	2.57	0.73^***^	0.05	0.10
Decision aids	0.93^***^	0.03	2.53	0.34^***^	0.04	0.09
Public health	0.84^***^	0.03	2.31	0.42^***^	0.04	0.11
Data sharing	0.77^***^	0.03	2.16	0.21^***^	0.04	0.09
Internet pharmacy	0.61^***^	0.03	1.83	0.09^*^	0.04	0.09
Monitoring & feedback	0.59^***^	0.03	1.80	−0.06	0.06	0.08
Customer eClaim	0.17^***^	0.03	1.18	−0.30^***^	0.04	0.18
Family doctor	0.02	0.03	1.03	−0.32^***^	0.04	0.12
Medical education	Ref			Ref		

a,b *Coefficient*.

b *SE, standard error*.

c *OR, odds ratio*.

d *SD, standard deviation*.

* *p < 0.05*;

**p < 0.01;

*** *p < 0.001*.

**Figure 2:**
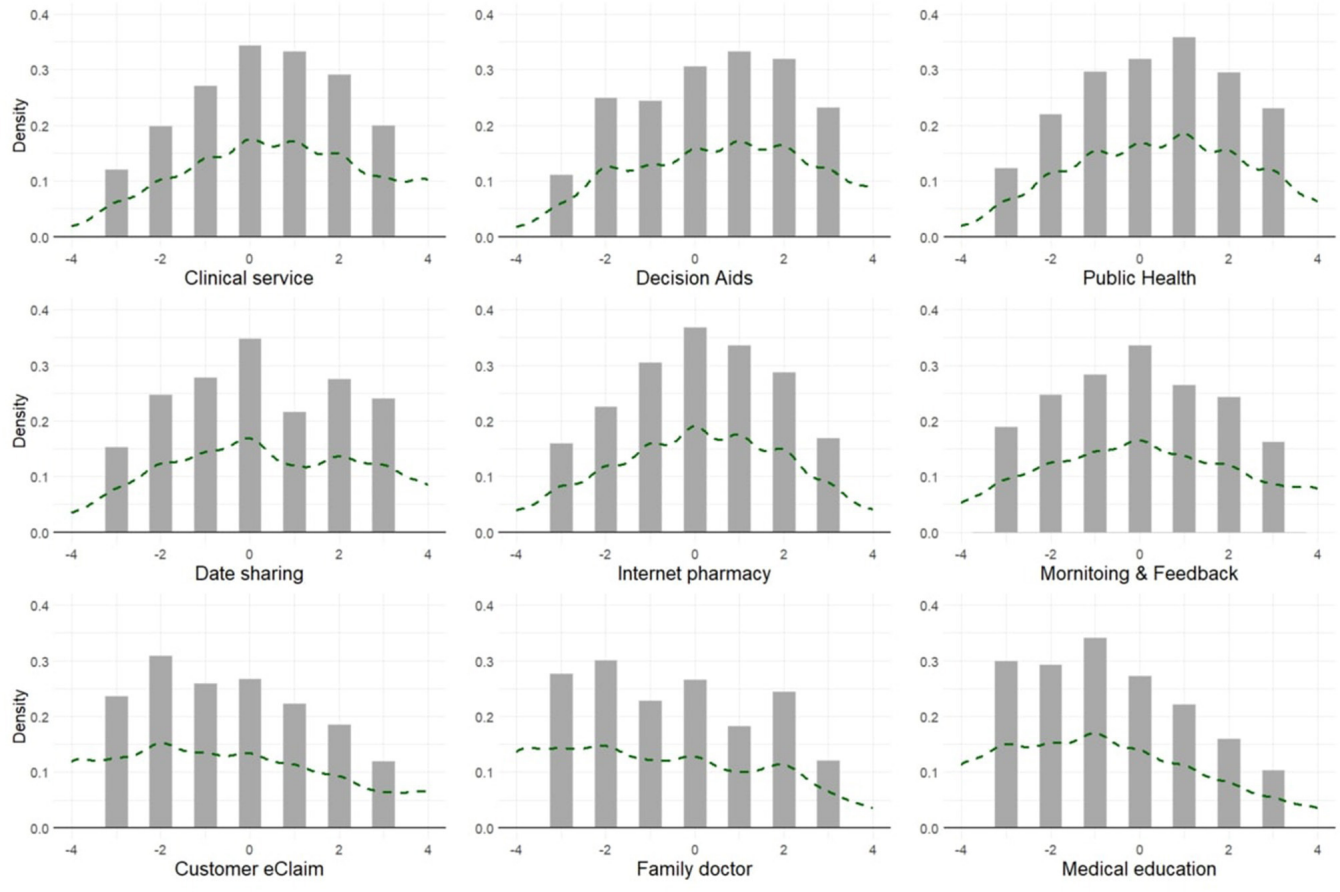
The score distribution of the BWS attributes.

Figure 2 presents the distribution and mean of BWS scores for each attribute. The scores for “Clinical Services,” “Decision Aids,” and “Public Health” showed negative skewness, whereas the scores for “Customer eClaim,” “Family Doctor,” and “Medical Education” showed positive skewness. The scores for “Data Sharing” had two peaks, indicating a bimodal distribution.

Stratified analyses showed that, compared with the other attributes, “Decision Aids,” “Internet Pharmacy,” and “Monitoring & Feedback” were more important from the senior students' perspective, whereas “Customer eClaim” and “Family doctor” were seen as more significant by junior students. Students studying non-clinical programs rated “Public Health” as more important than others; however, students studying clinical medicine indicated that “Medical Education” was more important (Table 6).

**Table 6:** Importance rank of mean BWS scores stratified by study years and study program.

	**Senior vs. Junior student^a^**		**Clinical vs. Non-clinical^b^**	
	**Δ^c^**	**95% C.I.^d^**	**Δ**	**95% C.I**.
Clinical service	0.08	−0.14 to 0.32	0.19	−0.04 to 0.42
Decision aids	0.32^**^	0.09 to 0.55	0.18	−0.05 to 0.40
Public health	−0.12	−0.34 to 0.11	−0.31^**^	−0.53 to −0.09
Data sharing	−0.22	−0.46 to 0.04	−0.21	−0.45 to 0.02
Internet pharmacy	0.33^**^	0.11 to 0.56	−0.21	−0.43 to 0.01
Monitoring &	0.32^*^	0.06 to 0.57	0.07	−0.17 to 0.32
feedback				
Customer eClaim	−0.47^**^	−0.74 to −0.32	−0.23	−0.49 to 0.28
Family doctor	−0.28^*^	−0.54 to −0.02	−0.1	−0.36 to 0.15
Medical education	0.01	−0.23 to 0.25	0.64^***^	0.41 to 0.88

a *Junior: Undergraduate student year 1~year 2; Senior: Undergraduate students year 3~year 5*.

b *Clinical: Students studying clinical programs, including Chinese medicine, Clinical medicine, Dentistry and Nursing*.

*Non-clinical: Students studying non-clinical programs, such as public health*.

c *Δ, difference in mean BWS score*.

d *95% C.I., 95% confidence interval*.

* *p < 0.05*;

** *p < 0.01*;

*** * p < 0.001*.

## Discussion

### Principal Findings

Internet-based healthcare services have been the source of a growing number of discussions in the last few years, especially with the recent outbreak of the COVID-19 pandemic, which has made accessing person-to-person healthcare services difficult. This study uses the BWS method to investigate medical students' preferences in ranking Internet-based healthcare services in China by importance We found that “Clinical Service,” “Decision Aids,” and “Public Health” were consistently ranked as the three most important health-related services that should be offered via the Internet based on the perspective of medical students who are soon to become healthcare service providers. This provides useful information to upgrade the current professional medical training system, which could be beneficial for doctors in preparing for the challenges of the proliferations of online healthcare information and for government in allocating resources to improve the effectiveness of online healthcare services.

### Comparisons With Previous Studies

As expected, the importance of offering clinical services through the Internet is noteworthy. Despite no studies investigating medical students' perspectives on online healthcare services were being found, an increasing number of studies have highlighted that patients with geographical or socioeconomic barriers inhibiting their access to high-quality healthcare resources are encouraged to use Internet-based healthcare services. For example, in the United Kingdom, the application of Internet videoconferencing with patients is encouraged in routine healthcare practice and it has proved to be an efficient and cost-effective way to provide primary and chronic-condition care (38, 39). However, there are some concerns that using online clinical services may result in regulatory and logistic uncertainties and may not be acceptable to doctors due to considerations such as safety, ethics, and patient experience (39, 40).

Online consultation is currently popular in China. The leading online medical service provider in China, Good Doctor Online, reported that at the end of 2019 it had 610,000 registered doctors providing online consultations via its platform. Few of them, however, had received standard training on how to provide such consultation effectively. Our findings demonstrated that medical students showed a positive attitude toward providing online healthcare services. It would therefore be beneficial to provide them with the necessary knowledge and skills to improve the effectiveness of Internet-based clinical services for patients and the methods that best employ online consultation alongside traditional clinical services. Moreover, emphasis should be placed on training medical students to provide standardized medical advice to patients through the Internet and on improving their adaptability and ability to provide online clinical services.

It is surprising that the provision of online medical training was identified in our survey as one of the least important services. In recent years, several colleges and universities have begun launching online curriculums in medical education worldwide (41), but our findings demonstrate that Chinese medical students may have low expectations of the quality and impact of online courses. Previous studies have found that insufficient digital literacy (42), a lack of time and effort to commit to online study (43), and difficulty in controlling the learning progress (24) can limit the use of Internet-based medical education. However, medical training to educate students in providing web-based services is rarely provided, despite the fact that that most people (95% of our respondents, for example) use the Internet to search for health information to some extent and that online medical education has several merits. This suggests that substantial demand for such courses could be generated by establishing a clear rationale for their usefulness and by providing appropriate and engaging curriculums containing innovative and well-designed content (25).

Web-based services, such as the online chronic disease self-management system (44), online pregnancy monitoring (45), and integrated infectious disease analysis platform (46), have reshaped the public health system in recent years. The World Health Organization has also confirmed that Internet techniques provide accessible, flexible, and rapid solutions that facilitate the development of health promotion, prevention, and treatment policies, as well as interventions aimed at reducing public health problems (47). Our findings demonstrated that a growing number of medical students have become aware of the importance of the links between public health and individual care, while further scrutiny of the preference heterogeneity data found that students studying non-clinical medicine ranked the provision of Internet-based public health services more highly than did those studying clinical medicine. Given that the COVID-19 outbreak may accelerate the process whereby public health services are migrated to the Internet, medical training programs should be prepared for this change. In addition, a difference between senior and junior medical students in service preference can be observed. For example, senior students considered the provision of online “Decision Aids” more important than did junior students. This is in line with previous findings that students with clinical experience better understanding the importance of providing information for supporting patient decision making by patients (48).

Our study used a choice-based method to provide important insights for developing a system of Internet-based healthcare services from the perspective of medical professionals. However, although medical students will be clinical professionals in the near future and more than half of the participants in our survey had working experience, currently, most of them were not yet qualified as doctors. Their limited knowledge of healthcare, their ability to understand some health issues, and their skills in navigating the healthcare system may affect their judgment and preferences in determining the importance of offering Internet-based healthcare services. In any reshaping of the role of the Internet in healthcare the preferences of medical professionals, patients, and other stakeholders need to be taking into consideration to improve patient care. We suggest the development an e-learning center would not only provide medical students with knowledge and skills in clinical practice, but also in the epistemology of “big health,” training medical students to shift from an individual and biomedical paradigm to a recognition of social and structural determinants of health and health equity (49). Using the resources and opportunities offered by the Internet could make this shift possible.

Our BWS questionnaire was developed based initially on the Chinese government's political documents and decree. However, the findings from a global literature review and focus group interviews assisted us in modifying these attributes to cover the most important aspects of Internet-based healthcare services [e.g., Online Medical Education (24–26) and Decision Aids (27, 28)]. The BWS method offers a simple, transparent, and people-centric approach to evaluate the relative importance of attributes that guide decisions, in a manner superior to conventional ranking methods. This method is feasible for both educators and researchers, and strengthens their confidence in informing the resources allocation processes by determining the priorities among a list of possibilities (21). Further studies should focus on dividing Internet-based services into specific components, such as features, functionalities, durations, modes of delivery, and cost, to investigate people's preferences regarding the different combinations of these components using BWS Case 2 or Case 3 methods and then determining the best solution to provide effective services to people with different health conditions or socioeconomic status.

## Limitations

In addition to the point made earlier regarding our respondents' relative inexperience and lack of intimate knowledge of the healthcare system, a number of other limitations have to be acknowledged. First, to reduce the cognitive burden on respondents, only nine types of services were included in the survey, and other Internet-related services (e.g., provision of traditional Chinese Medicine and applying artificial intelligence in healthcare) were not included. Second, the BWS Case 1 approach is a parsimonious method and the trade-offs between different levels of preference could not be evaluated. Third, in this study, we used a convenient sample of students from two medical universities in one province, most aged 25 years or less, which might interfere with the ability to generalize our results to the general public. Lastly, the data we used in the analyses came from an online survey. As such, the possibility of information and selection bias may arise, for example, as a result of certain types of medical students opting not to engage in the survey process.

## Conclusions

This study using a BWS method quantified the heterogeneities in medical students' preferences in relation to Internet-based healthcare services. Based on their perspective, providing “Clinical Service,” “Decision Aids,” and “Public Health” through the Internet are the three most important services. An extension of this research would be to better define the content and style of each type of Internet-based service that would be appropriate to different stakeholders, and provide empirical evidence to shape a nation-wide Internet-based healthcare services system.

## Data Availability Statement

The raw data supporting the conclusions of this article will be made available by the authors, without undue reservation.

## Ethics Statement

The studies involving human participants were reviewed and approved by the Institutional Review Board of the Third Affiliated Hospital of the Southern Medical University. The patients/participants provided their written informed consent to participate in this study.

## Author Contributions

RX: conceptualization, methodology, software, formal analysis, visualization, data curation, writing—original draft preparation, and writing—review and editing. L-mZ: conceptualization, investigation, resources, and writing—review and editing. EW: conceptualization, validation, supervision, and writing—review and editing. DW: conceptualization, validation, resources, supervision, writing—review and editing, project administration, and funding acquisition. All authors contributed to the article and approved the submitted version.

## Funding

This study was funded by a grant from the Natural Science Foundation of Guangdong Province (Ref: 2021A1515011973), Philosophy and Social Sciences of Guangdong College for the project of Public Health Policy Research; and Evaluation Key Laboratory (2015WSYS0010), and Public Health Service System Construction Research Foundation of Guangzhou, China (2021-2023).

## Conflict of Interest

The authors declare that the research was conducted in the absence of any commercial or financial relationships that could be construed as a potential conflict of interest.

## Publisher's Note

All claims expressed in this article are solely those of the authors and do not necessarily represent those of their affiliated organizations, or those of the publisher, the editors and the reviewers. Any product that may be evaluated in this article, or claim that may be made by its manufacturer, is not guaranteed or endorsed by the publisher.

## Abbreviations

BIBD: Balanced incomplete block design
BWS: Best-Worst Scaling
MNL: Multinomial Logit
MXL: Mixed Logit.
